# Polymorphisms in RYBP and AOAH Genes Are Associated with Chronic Rhinosinusitis in a Chinese Population: A Replication Study

**DOI:** 10.1371/journal.pone.0039247

**Published:** 2012-06-19

**Authors:** Yuan Zhang, Leandra Mfuna Endam, Abdelali Filali-Mouhim, Liping Zhao, Martin Desrosiers, Demin Han, Luo Zhang

**Affiliations:** 1 Department of Otolaryngology Head and Neck Surgery, Beijing TongRen Hospital, Capital Medical University, Beijing, People’s Republic of China; 2 Key Laboratory Otolaryngology Head and Neck Surgery, Ministry of Education of China, Beijing Institute of Otolaryngology, Beijing, People’s Republic of China; 3 Department of Otolaryngology, Centre de Recherche du Centre Hospitalier de l’Université de Montréal (CRCHUM), Montreal, Quebec, Canada; 4 Department of Otolaryngology-Head and Neck Surgery, Montreal General Hospital, McGill University, Montreal, Quebec, Canada; Centro de Pesquisa Rene Rachou/Fundação Oswaldo Cruz (Fiocruz-Minas), Brazil

## Abstract

**Background:**

The development of CRS is believed to be the result of combined interactions between the genetic background of the affected subject and environmental factors.

**Objectives:**

To replicate and extend our recent findings from genetic association studies in chronic rhinosinusitis (CRS) performed in a Canadian Caucasian population in a Chinese population.

**Methods:**

In a case-control replication study, DNA samples were obtained from CRS with (n = 306; CRSwNP) and without (n = 332; CRSsNP) nasal polyps, and controls (n = 315) in a Chinese population. A total of forty-nine single nucleotide polymorphisms (SNPs) selected from previous identified SNPs associated with CRS in Canadian population, and SNPs from the CHB HapMap dataset were individually genotyped.

**Results:**

We identified two SNPs respectively in RYBP (rs4532099, p = 2.15E–06, OR = 2.59) and AOAH (rs4504543, p = 0.0001152, OR = 0.58) significantly associated with whole CRS cohort. Subgroup analysis for the presence of nasal polyps (CRSwNP and CRSsNP) displayed significant association in CRSwNP cohorts regarding to one SNP in RYBP (*P* = 3.24^E^–006, OR = 2.76). Evidence of association in the CRSsNP groups in terms of 2 SNPs (AOAH_rs4504543 and RYBP_rs4532099) was detected as well. Stratifying analysis by gender demonstrated that none of the selected SNPs were associated with CRSwNP as well as CRSsNP. Meanwhile 3 SNPs (IL1A_rs17561, P = 0.005778; IL1A_rs1800587, P = 0.009561; IRAK4_rs4251513, P = 0.03837) were associated with serum total IgE level.

**Conclusions:**

These genes are biologically plausible, with roles in regulation of transcription (RYBP) and inflammatory response (AOAH). The present data suggests the potential common genetic basis in the development of CRS in Chinese and Caucasian population.

## Introduction

Chronic rhinosinusitis (CRS) is a common inflammatory disorder of the sinus and paranasal sinus mucosa with a highly heterogeneous pathogenesis. Because of its negative impact on patients’ quality of life and the concomitant increasing social economic burden, CRS has become a global health problem [1]. The development of CRS is believed to be the result of combinations between the genetic background of the affected subject and environmental factors [1], [2]. However, there is still no clear answer as to their exact contributions to the process and the mechanisms of pathogenesis of CRS.

A genetic basis to sinusitis is strongly suggested by clinical and experimental evidence [3]–[10]. In humans, genetic disorder such as cystic fibrosis and primary ciliary dyskinesia [11] are frequently associated with development of sinusitis in knockout mouse models, deletion of several genes leads to development of sinusitis.

It has been demonstrated that single nucleotide polymorphisms (SNPs) in several genes are associated with CRS [3]–[10] These are biologically plausible, with roles in regulation of transcription (Ring1A and YY1 binding protein, RYBP) [12]_ENREF_13, inflammatory response (acyloxyacyl hydroxylase, AOAH) [13] and innate immune response (IL1RL1 [5], [14] and interleukin-1 receptor-associated kinase 4 (IRAK4) [6], [15]), are associated with chronic rhinosinusitis (CRS) in a Canadian Caucasian population [16]. Replication of results of a genetic disease association study in independent samples has emerged as a standard for demonstrating the relevance of a candidate gene for a complex trait.

Given the evidence above, genetic backgroung plays potencial roles in the development of CRS and we hypothesized genes which were demontrated as susceptible genes for CRS in Caucasian population also exerted effects in Chinese cohort. Therefore, the aim of this study was to replicate polymorphisms in the genes performed in Canadian Caucasian population previously are associated with the Chinese population. A population-based case-control association analysis was used to assess the risk of CRS conferred by SNPs in the candidate genes in our Han Chinese cohort.

## Materials and Methods

### Study Subjects

306 CRS with nasal polyps (CRSwNP subjects) (180 males and 126 females) and 332 individuals affected with CRS without nasal polyps (CRSsNP) (190 males and 142 females) were prospectively recruited from the rhinology ward of Beijing Tongren Hospital between February 2008 to July 2009. A total of 315 healthy controls, of which 146 (46.3%) were female, were recruited as well. All the subjects were of Chinese Han ethnic origin and all from the north region of China. The study was approved by the Beijing Tongren Hospital Ethics Committee, and written informed consent was obtained from all participants.

Diagnosis of CRSwNP and CRSsNP was based on American Academy of Otolaryngology-Head and Neck Surgery (AAO-HNS) 2004 guidelines [17], based on assement by a single ENT doctor specialized in sinus diseases. All the CRS cases recruited in present study were unresponsive to all forms of medical therapies such as topical or intranasal corticosteroid and long term-low dose antibiotic or presented persistent signs/symptoms of CRS despite previous endoscopic sinus surgery (ESS). Patients were interviewed by trained personnel, and a standardized questionnaire was used to obtain items including demographic variables and personal and familial antecedents of allergies. Patients also underwent a standard set of laboratory tests that included measurements of total IgE to assess allergic status. Controls were recruited following two strategies: either spouses or non-blood relatives living in the same household and individuals recruited from a geographic area similar to that of CRS patients. The only attempt at matching subjects and control is their geographical location to minimize differences secondary to differences in potential environmental exposures. Nevertheless, a standardized questionnaire assessing age, sex and ethnic origin (but not smoking, history of atopy or physician diagnosed asthma) was obtained for controls. Moreover, all the controls showed negative of serum phadiatop determination.

### SNP Selection

**Figure 1:**
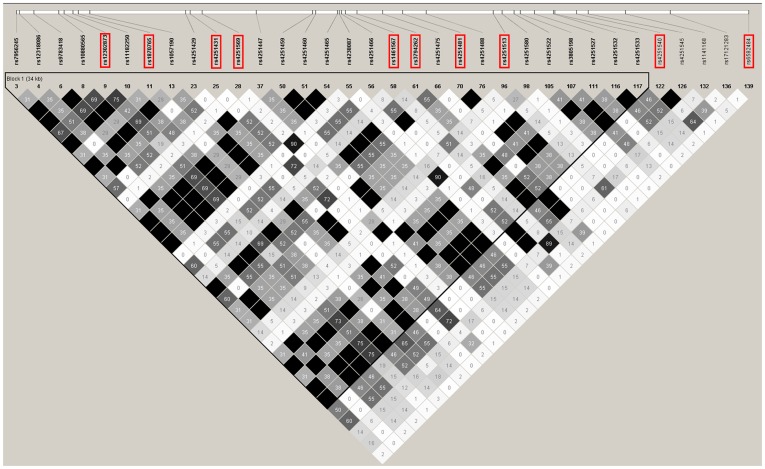
Linkage Dysequilibrium (LD) plot for IRAK4. The LD plots were generated by Haploview 4.1. The white horizontal bar below the info track illustrates the location of SNPs on a physical scale. The shade of squares illustrates the strength of pairwise r2 values on a black and white scale where black indicates perfect LD (r2 = 1.00) and white indicates perfect equilibrium (r2 = 0). The r2 LD value is also indicated within each square. Failed SNP and SNPs not in Hardy-Weinberg equilibrium or with low minor allele frequency are not illustrated.

A total of forty-one single nucleotide polymorphisms (SNPs) selected from previous identified associated with CRS in Canadian population [3], [5]–[7], [9], [10], [16], [18], [19] were choose for genotying (Table 1). In addition, SNPs in IRAK4 gene from the CHB HapMap dataset were also individually genotyped. Briefly, the International Haplotype Mapping (HapMap) (www.hapmap.org) SNP databases were used to select SNPs in the IRAK4 gene region. The screened region was extended 10 kilobases upstream of the annotated transcription start site and downstream at the end of the last IRAK4 exon. The SNPs were selected to extract the most genetic information based on CHB haplotype data using the HAPMAP database (Hapmap Data Rel 27 Phase II+III, Feb09) [20]. From this dataset, 34 SNPs in IRAK4 gene region were selected using a pairwise tagging algorithm implemented in Haploview version 4.1 program [21]. In addition, when we set Hardy-Weinberg p value cutoff, minor allele frequency and r2 thresholds at 0.01, 0.05 and 0.8, respectively, the LD pattern for IRAK4 gene in our population showed strong LD in several groups of SNPs, indicating that the SNPs in each group represent a common region (Figure 1). Consequently, we choose 10 SNPs including rs4251513, rs1461567, rs3794262, rs4251481, rs4251540, rs4251569, rs6582484, rs4251431, rs1870765 and rs12302873 to represent the entire 34 loci for eventual genotyping and the former two SNPs were composed in the selected SNPs from the previous identified associated with CRS in Canadian population. Therefore, 49 SNPs constituted the selection set to be genotyped in our patient and controls eventually.

**Table 1:** SNPs selected from previous identified associated with CRS in Canadian population.

Chromosome	Gene	SNP
1	PARS2	rs2873551
	IL22RA1	rs4292900; rs4648936; rs16829225
	TNFRSF1B	rs235214; rs496888; rs652625; rs7550488
2	TRIP12	rs1035833
	IL1RL1	rs13431828; rs10204137
	IL1A	rs17561; rs2856838; rs2048874; rs1800587
3	FAM79B	rs13059863
	RYBP	rs4532099
5	TSLP	rs3806932;rs2289276
6	LAMA2	rs2571584
	TNFAIP3	rs3757173; rs5029938
7	LAMB1	rs4727695
	AOAH	rs4504543
	MET	rs38850
	RAC1	rs836479
	CACNA2D1	rs6972720
8	KIAA1456	rs11779957
	MSRA	rs7001821
9	MUSK	rs10817091
11	PDGFD	rs12574463
12	NOS1	rs1483757
	NAV3	rs1726427
	IRAK4	rs4251559; rs4251513; rs146567
14	SERPINA1	rs1243168; rs4900229
15	UBE3A	rs1557871
20	SLC13A3	rs393990
22	CACNA1I	rs3788568

### Genotyping

DNA was collected in EDTA-treated tubes and isolated from peripheral blood leukocytes, using the DNA Isolation Kit for Mammalian Blood (Roche, Indianapolis, USA). Isolated DNA from blood was stored at 4°C prior to use. To reduce genotyping cost, the majority of the selected SNPs were genotyped by the MassArray system (Sequenom) with primers and probes (Table 2) as described. One SNP (rs12302873) which was evaluated by preliminary test unsuitable to be genotyped through MassArray approach were identified by direct sequencing of PCR products of genomic DNA (Table 3). Genotyping was performed without knowledge of the case or control status. A 10% random sample was tested in duplicate by different persons, and the reproducibility was 100%.

**Table 2:** Details of the primers used in the screening of SNPs by MassArray.

Gene	SNP	Primers (5′ - 3′)	Extension Primers (5′ - 3′)
PARS2	rs2873551	ACGTTGGATGCAAACCACTTACAAGGTGGG	CACGAGTGTCTCACCAA
		ACGTTGGATGAATTACTTGCCCTGTGTGCC	
IL22RA1	rs4292900	ACGTTGGATGCCTTCCGACTTGCAGAAAAC	ACTTGCAGAAAACAGCAATAG
		ACGTTGGATGACCACTTGGGATGAATCAGC	
	rs4648936	ACGTTGGATGCTGGAGTCAGCCTAAGATTG	ttggaCCGGTGTGTGCAGCGCGAG
		ACGTTGGATGCCTTAGGAGATTGTCAAGGG	
	rs16829225	ACGTTGGATGGATGAAGATTCAGGCTGCTC	AGGCTGCTCTCCCATCATTTTG
		ACGTTGGATGTCCCTTCATTCACACGAAGC	
TNFRSF1B	rs235214	ACGTTGGATGAAAAGCAAGGTGTTGCCAGG	TTGCCAGGCCTGCTAGGCTCAAA
		ACGTTGGATGCAAGGAATCAGATTCTCCCC	
	rs496888	ACGTTGGATGTCTCAAACCCACTGCTTGAC	GCCTGGTCTTAGGACAC
		ACGTTGGATGATATCCTGACCCCACAGCCT	
	rs652625	ACGTTGGATGTCTAGTTGTCCCCCACACAC	ACACCTCAAGACCAATGGG
		ACGTTGGATGATAGGGAAACTGGCAGGAGG	
	rs7550488	ACGTTGGATGTCCCAGCCTTTAGATTCACC	CTGCTCAGCCCAACCTCC
		ACGTTGGATGGGGTAGACATCTTTCTGGG	
TRIP12	rs1035833	ACGTTGGATGTCGCTGTCCTGTTTTTATGC	TCTTCCATTCTTACATGATCT
		ACGTTGGATGGATGTGTATCTCAGATTACC	
IL1RL1	rs13431828	ACGTTGGATGCGTTGTTGAGATTACTCCAG	ggAGATGAGTCACTGGCATAC
		ACGTTGGATGAGAGTATCACCAACTGCCTC	
IL1A	rs17561	ACGTTGGATGTCACATTGCTCAGGAAGCTA	ATTGCTCAGGAAGCTAAAAGGTG
		ACGTTGGATGATCTGCACTTGTGATCATGG	
	rs1800587	ACGTTGGATGTGGGAGAAAGGAAGGCATGG	GGATTTTTACATATGAGCCTTCAATG
		ACGTTGGATGGGCCACAGGAATTATAAAAGC	
FAM79B	rs13059863	ACGTTGGATGTGATTGACAGGAGTCATGGG	GGTGAGTGGTTAAGGATAG
		ACGTTGGATGACTGGCACTATGTTAAACAC	
LAMA2	rs2571584	ACGTTGGATGTAAATCTGGGCAGTTGAGGG	CAGTTGAGGGATTGCTTTTAACAGAA
		ACGTTGGATGTAGTATCTATATCCCCTGTC	
TNFAIP3	rs3757173	ACGTTGGATGTCAGATGGAAAGAGATGGGC	GGCAGTAGGAAGATTTTAAACAAA
		ACGTTGGATGAGAGTCAGGCAAGCAAAAAG	
	rs5029938	ACGTTGGATGCTCTTGTGAAATGAGGGCAG	ATGAGGGCAGTAAGTGAT
		ACGTTGGATGGCCTTCACCAGCAAATCAAG	
LAMB1	rs4727695	ACGTTGGATGTCCCTACTGTTCCATTTCTC	CCATTTCTCTTTATTTCCATCTC
		ACGTTGGATGTATTCTCACCACTGAGCCAC	
MET	rs38850	ACGTTGGATGGGCTACTACACTTAACCATT	ACACTTAACCATTATGTAACTTC
		ACGTTGGATGCCTGAGATGCAGAAGGTGTT	
RAC1	rs836479	ACGTTGGATGTGGGTTTGGTTTGTTTCCCG	CGCCTTCCTCCTTGTGC
		ACGTTGGATGCTGCCACACCAGCAAATGTC	
KIAA1456	rs11779957	ACGTTGGATGCATAATCACAACTTAAAGGC	TTAAAGGCAAAAGTAGTACTC
		ACGTTGGATGGGCTCTCAGCAGGAAAATAC	
MSRA	rs7001821	ACGTTGGATGTGAGTTGATCGATCTAGAGG	TCTAGAGGTTAATGTATTATAAAGAA
		ACGTTGGATGACTCACCAGCCTCCATAATC	
MUSK	rs10817091	ACGTTGGATGCTATTCAAAACCTATTGTC	TATTTTGCATTATATACTTAATGCT
		ACGTTGGATGTCTGCTAGTATTGAATCCTC	
PDGFD	rs12574463	ACGTTGGATGAGGAGAGTGATGCCAAACAG	AGTTTACATCCAAACTATAGAGG
		ACGTTGGATGATGGTGCAGTCTCATAACTC	
NOS1	rs1483757	ACGTTGGATGCAACTGAGCTGATTCTCTGG	ggTGGGGTTGAAATTGACTTCC
		ACGTTGGATGAAAGGGACACTAGGCAAGAG	
NAV3	rs1726427	ACGTTGGATGATCTATGACTTGCACAGGAG	tCACAGGAGTTGTGTAGC
		ACGTTGGATGAGCACCTGGCACTTTATTGG	
IRAK4	rs4251559	ACGTTGGATGGATACAGTTGGTGGTACAGG	GGTACAGGCAATAAGTAAAACA
		ACGTTGGATGCCTGTTGCCCCTTTCTTTAG	
SERPINA1	rs1243168	ACGTTGGATGTGCCTGTGAATAATCCAACC	ATAATCCAACCAAGAGCAACACAAA
		ACGTTGGATGACAGATGACCTCAAACACCC	
	rs4900229	ACGTTGGATGAGAACTCCTCACCCAGCAGA	GGGTTGTGCAGAGAGGCT
		ACGTTGGATGACGAGAAGCCCTGAGAGTG	
UBE3A	rs1557871	ACGTTGGATGCCCACACCTGCATCAAAATC	AATCTTGCAGTGCCTATTAA
		ACGTTGGATGAGTCCCAAGTCTTTTCTCCC	
SLC13A3	rs393990	ACGTTGGATGTAGGTGGAGCTGCTCTATTC	ggaCCAGTGAAAATAATTGTCATG
		ACGTTGGATGTGTCCTGCATGTGGAGTTTC	
CACNA1I	rs3788568	ACGTTGGATGCTTATGCCTGACATGGCACC	gCACTCGGGGGAGATGGAC
		ACGTTGGATGGCCTTCAGAACAAAGAGACC	

**Table 3:** Details of the primers used in the screening of SNPs by PCR resequencing.

Gene	SNP	Primers (forward)	Primers (reverse)
IL1RL1	rs10204137	CCCCTCAGATCACTCACAAT	AGCCAGCTAGGAGAAGTCAG
IL1A	rs2856838	TGGGACTGCTATTCTTACAC	CTTTCCAATTAGTTCCCTCT
	rs2048874;	TGTGGAGGGGCAGTCATA	ACCAACACCAGCAGTATA
RYBP	rs4532099	CTGTGAAGGTGGAAATACTGT	GAAATGTCAAGAAGTTTACGG
AOAH	rs4504543	CAACATCAGCCTACAGAA	CTTTCTCCTTTCTTACCA
CACNA2D1	rs6972720	ACTACTGGTTTCCTTGCTCC	CCTTCCTTCCAGTACATCTCAA
IRAK4	rs12302873	AATGTGGCATACACCTACC	GTTGTGAATAGTTGGAGGC

### Determination of Serum total IgE and Allergen-specific IgE

Serum total and allergen-specific IgE were quantified using Phadiatop test which is based in immunoCap 100 system according to the manufactures’s directions (Pharmacia, Uppsala, Sweden). The allergen-specific IgE phadiatop covered all the common aeroallergens which included Dermatophagoides pteronyssinus (Der p); Dermatophagoides farinae (Der f); Animal hair; Trees; Grasses; Cereals; Mugwort; Dandelion; Giant ragweed; Chenopodium album; Humulus; Locust; Blatella germanica; Pine; Plantain; Curvularia lunata; Candida albicans; Penicillium notatum; Alternaria tenuis and Aspergillus fumigatu. The CAP classification system divides results into seven categories from 0 to 6. Additional classes are scored as follows: 0.35–0.70 kU/L, class 1; 0.71–3.5 kU/L, class 2; 3.51–7.5 kU/L, class 3; 7.6–17.5 kU/L, class 4; 17.6–50 kU/L, class 5; 50 kU/L, class 6. The units reported by CAP are in accordance with the defined WHO serum standard IRP 75/520. For the present analyses, subjects were considered as sensitive to the allergens if the measurement of allergen-specific IgE phadiatop was equal to or above 0.35 kU/L.

### Statistical Analyses

PLINK program version v1.02 was used to determine association. The association test is based on comparing allele frequencies between cases and controls using Chi-squared tests (χ2). We estimated odds ratios (OR) and 95% confidence intervals (95% CI) for the effect of polymorphisms on CRS risk. A corrected p-value of <0.05 was considered statistically significant. Bonferroni correction over the tested SNPs was performed for multiple adjustments. Subanalysis restricted to the presence of nasal polyps was also performed to examine whether the effect of observed associations within the population differed within the subgroups. Associations between genotype and IgE levels for all patients were assessed using an Anova test, which was performed in the *R* statistics software version 2.3.1. Haploview 4.1 software was used to generate the linkage disequilibrium (LD) plot.

## Results

### Population Characteristics

Table 4 provides a summary of the demographic characteristics of the study population. Age and gender were all well-balanced between cases and controls. The cohort of 306 CRSwNP patients had a mean age of 43 years and consisted of slightly more men (58.8%) than women (41.2%), while the 332 CRSsNP individuals had a mean age of 39 years and also consisted of slightly more men (57.2%). For the 315 healthy controls mean age were 36, with 53.7% men and 46.3% women. 14.1% and 17.8% individuals were atoptic as demonstrated serum Phadiatop positive results in CRSwNP and CRSsNP cases respectively. All the subjects lived in an urbainised region the north of China and the majority of each study group belonged to Beijing and Hebei.

**Table 4:** Demographic characteristics of the study population.

Characteristic	CRSwNP (n = 306)	P (vs. Controls)	CRSsNP (n = 332)	P (vs. Controls)	Controls (n = 315)
Age Mean (Range) (years)	43±16 (7–77)	0.0618	39±16 (7–77)	0.0506	36±15 (3–78)
Sex, No.(%) of Male	180 (58.8)	0.194	190 (57.2)	0.360	169 (53.7)
Total IgE, kU/l	120.2±211.4	0.0006*	112.1±277.1	0.0183*	57.4±111.9
Serum phadiatop +, No.(%)	43 (14.1)	0.200#	59 (17.8)	-	-
Living city, No.(%)					
Beijing	217 (70.9)		233 (70.2)		146 (57.0)
Hebei	25 (8.2)		29 (8.7)		19 (19.2)
Others	64 (20.9)		70 (21.1)		46 (23.8)

* : P value <0.05

# : P-value regarding to serum phadiatop between CRSwNP and CRSsNP subjects.

### Association Analysis

Allele frequencies for all 49 SNPs were calculated and the significant associations between alleles and CRS phenotype were shown in Table 5. The significant associations (P_<_0.05) only existed among the genes coding RYBP (rs4532099), AOAH (rs4504543) and IRAK4 (rs1461567, rs4251559 and rs3794262) genes at 5 loci. Table 5 shows odds ratios for risk allele and the corresponding P values. Two SNPs respectively in RYBP (rs4532099, P = 2.15E–06, OR = 2.59) and AOAH (rs4504543, P = 0.0001152, OR = 0.58) remained significant following application of the Bonferroni correction for multiple testing for 49 simultaneous tests (P<0.001).

As for the subgroup analysis for the presence of nasal polyps (CRSwNP and CRSsNP) displayed significant association in CRSwNP cohorts regarding to one SNP in RYBP (rs4532099) and 5 SNPs IRAK4 (rs4252431, 6582484, rs1461567, rs4251559 and rs3794262) (Table 6). Among the six SNPs, only rs4532099 in RYBP (P = 3.24E–006, OR = 2.76) remained significant following application of the Bonferroni multiple testing (P<0.001). Likewise, we detected evidence of association in the CRSsNP subgroups in terms of 4 SNPs (AOAH_rs4504543, RYBP_rs4532099, IRAK4_rs1461567 and IL1RL1_rs13431828) as well (Table 6), while only rs4504543 in AOAH (P = 8.11E–011, OR = 0.30) and rs4532099 in RYBP (P = 4.12E–005, OR = 2.45) remained significant following application of the multiple adjustment (P<0.001).

In order to verify potential association between total serum IgE levels and the selected SNPs, a quantitative trait analysis was performed. As presented in Table 7, 3 SNPs (IL1A_rs17561, P = 0.005778; IL1A_rs1800587, P = 0.009561; IRAK4_rs4251513, P = 0.03837) were associated with serum total IgE level.

**Table 5:** Single nucleotide polymorphisms associated with chronic rhinosinusitis.

SNP	Associate allele	Case; Control Frequencies	Chi squared	OR	P
RYBP_rs4532099	A	0.14; 0.06	22.46	2.59	2.15×10^−6^ *
AOAH_rs4504543	C	0.13; 0.21	14.87	0.58	0.0001152*
IRAK4_rs1461567	C	0.48; 0.54	5.36	0.79	0.0206
IRAK4_rs4251559	A	0.45; 0.51	5.331	0.79	0.02095
IRAK4_rs3794262	T	0.14; 0.18	4.998	0.73	0.02538

SNP: Single nucleotide polymorphisms; OR: Odd ratio; P: p-value.

* : P value remains significant after Bonferroni correction.

**Table 6:** Single nucleotide polymorphisms associated with subgroups of CRSwNP and CRSsNP.

SNP	Associate allele	CRSwNP				CRSsNP			
		Case; Control Frequencies	Chi squared	OR	P	Case; Control Frequencies	Chi squared	OR	P
RYBP_rs4532099	A	0.15; 0.06	21.67	2.76	3.24×10^−6^ *	0.13; 0.06	16.82	2.45	4.12×10^−5^ *
IRAK4_rs4251431	T	0.07; 0.11	6.07	0.60	0.01375	0.09; 0.06	0.94	0.91	0.3313
IRAK4_rs6582484	C	0.09; 0.13	5.154	0.65	0.02319	0.10; 0.13	0.70	0.93	0.4039
IRAK4_rs1461567	C	0.48; 0.54	4.284	0.79	0.03848	0.48; 0.54	4.453	0.79	0.03484
IRAK4_rs3794262	T	0.13; 0.18	4.157	0.72	0.04145	0.14; 0.18	2.05	0.89	0.1523
IRAK4_rs4251559	A	0.45; 0.51	3.852	0.79	0.04968	0.45; 0.51	2.86	0.91	0.0911
AOAH_rs4504543	C	0.20; 0.21	0.22	0.96	0.6371	0.07; 0.21	42.23	0.30	8.11×10^−11^ *
IL1RL1_rs13431828	T	0.10; 0.11	0.74	0.92	0.3887	0.07; 0.11	4.032	0.64	0.04464

SNP: Single nucleotide polymorphisms; OR: Odd ratio; P: p-value.

* : P value remains significant after Bonferroni correction.

**Table 7:** Association between polymorphisms and IgE levels.

SNP	BETA	SE	R2	T	P
IL1A_rs17561	58.15	21	0.01112	2.769	0.005778
IL1A_rs1800587	54.14	20.84	0.009762	2.599	0.009561
IRAK4_rs4251513	31.54	15.21	0.004459	2.074	0.03837

BETA: regression coefficient; SE: standard error; R2: regression r-squared; T: Wald test (based on t-distribtion); P: p-value.

## Discussion

In this study, we replicate a number of genes in CRS in previously identified in Caucasians in a Han Chinese population. Genes associated with CRS and the Caucasian population has not yet being replicated in the Chinese population and the replication suggests a common basis.

The genes identified are of potential important biological significance. RYBP is a regulator of transcription [12]. ILIRL1 is associated with Toll-like receptor (TLR) signaling regulation [14]. SNPs in the IL1RL1 gene have previously been shown to affect serum level of eosinophilia and IgE in other models [5]. IRAK4 also is implicated as a signaling intermediate in the TLR signaling pathway [16], [22], and SNPs in the IRAK4 gene have been documented to have a functional impact, within genotype specific effect on serum IgE level [6], [23].

Importance of TLR signaling is suggested by their role in detecting and regulating responses to gram-positive and gram-negative bacteria. Importance of IRAK-4 is suggested by the description of an enhanced susceptibility to infection with gram-positive bacteria in IRAK-4 deficient children. Previous work from the Desrosiers group has identified altered function of the TLR signaling system as key to the pathogenesis of CRS. Using complex-model analysis of pooling-based genome wide association testing on the Canadian population with CRS, they identified the polymorphisms at multiple levels of the TLR signaling cascade all confer an increased risk in CRS [2], [16]. Functional support for this concept has been provided by in vitro model of epithelial cell culture documenting reduced response to TLR agonists in epithelial cells from CRS patients [24].

The identification of the AOAH gene is also of potential significant interest and suggests a novel mechanism for the development of CRS, again implicating an innate immune signaling, but in a novel fashion. AOAH is responsible for degrading lipopolysaccharide (LPS), and dysfunctional AOAH gene function leads to decreased LPS degradation with unopposed continued LPS stimulation via a TLR-4 dependant mechanism. In AOAH knockout mouse models, persistent inflammation following LPS stimulation is observed [25]–[27]. Supporting a role in airway disease, the AOAH gene has previously been implicated in a genome wide scan for asthma [13]. Corresponding to the above literature, here we presented that rs4504543 in the AOAH gene played a protective role (OR = 0.58) in CRS with a strong P value (P = 0.0001152). Moreover, the AOAH_ rs4504543 loci was also revealed as a protective factor (OR = 0.30) in CRSsNP group with a stronger P value (P = 8.11–011), indicating that AOAH gene might exert a crucial protective role in the development of CRS.

RYBP is a zinc finger protein with an essential role during embryonic development, which binds transcriptional factors, polycomb products, and mediators of apoptosis, suggesting roles in apparently,unrelated functions. Gene products of the RYBP gene inhibit ubiquitination and subsequent degradation of TP53, and, by interacting with MDM2, play a role in regulating transcription of TP53 target genes and promoting apoptosis. Recent findings have also suggested that RYBP may also play a role in epigenetic regulation [28], and contribute to defense against retroviruses [29]. It is thus possible that polymorphisms in the RYBP gene may be implicated in CRS by dysregulating TP53 activity in TP53 or in its target genes via alteration of RYBP gene products or by altering binding at regulatory binding sites in the RYBP gene promotor area, which contains binding sites for the following transcription factors (YY1, IRF-1, C/EBPA, GATA-1, POU2F1). This may contribute to the inflammation observed in CRS, promoting epithelial dysfunction with secondary bacterial colonization. In contrast to AOAH gene, polymorphisms in the RYBP gene was exhibited here for the first time as a significantly risk factor of CRS and either of the subgroups (CRSsNP and CRSwNP) with high OR values (OR_CRS_ = 2.59; OR_CRSsNP_ = 2.76; OR_CRSwNP_ = 2.45), suggesting the variation of rs4532099 in RYBP could increase the risk of CRS development.

IL1RL1 gene is involved in regulation of TLR signaling and has recently also been implicated as the receptor for IL-33. Its role in development of CRS may be via interfering with TLR signaling, or via an alteration of IL-33 homeostasis.

Taken overall, all of the replicated genes have disparate functions, but evidence supports that dysfunctions in each these genes may conceivably contribute to development of CRS, underlining the concept that CRS represents a common morphological appearance of clinical disease as an endpoint of multiple unique pathogenic mechasnisms.

Our study has obvious limitations. First and most noticeable is the small group size of the sample used. Nevertheless, given the limited number of genes genotyped in this replication study, feet corrected P value remains significant and, in the case of the RYBP and AOAH genes, is highly significant with a high odds ratio. A second consideration is the ethnic variability in SNP frequency known for these genes. It is clear that in this study, we have replicated SNPs associated in a Caucasian population, and have not performed extensive fine mapping studies of the gene which might identify other risk SNPs. As shown in this example however, when gene coverage is adjusted to better reflect tagging SNP selection for the CHB data set, we are able to identify significant polymorphisms within the IRAK4 gene.

We replicate several genes which were proved to be associated with CRS in Caucasian population in a Chinese population, suggesting a common basis to the development of the disease in both population types. Of interest is the potential implication of inflammatory pathways, suggesting dysfunction in TLR signaling as a critical element in chronic rhinosinusitis. While these studies have significant limitations, they nevertheless offer a basis for further exploration of a role for immune signaling and response to bacteria in further studies of CRS.
